# Perception of the Current Anti-doping Regime – A Quantitative Study Among German Top-Level Cyclists and Track and Field Athletes

**DOI:** 10.3389/fpsyg.2018.01890

**Published:** 2018-10-16

**Authors:** Daniel Westmattelmann, Dennis Dreiskämper, Bernd Strauß, Gerhard Schewe, Jonas Plass

**Affiliations:** ^1^Center for Management, University of Münster, Münster, Germany; ^2^Institute of Sport and Exercise Sciences, Department of Sport Psychology, University of Münster, Münster, Germany; ^3^gekko mbH, Berlin, Germany

**Keywords:** anti-doping, performance enhancing drugs, policy, cycling, athletics, elite sport, deterrence theory

## Abstract

In recent years anti-doping organizations have implemented various measures to deter elite athletes from using performance-enhancing drugs. One of the main challenges in the fight against doping is that the effectiveness of these anti-doping measures is still unknown. Since the effectiveness of the measures depends primarily on the athletes’ perception, this study focuses on the following four objectives: (1) How effective do top-level athletes perceive individual anti-doping measures to be? (2) Are the results stable across different sports and (3) genders? (4) How can the anti-doping measures be structured into appropriate categories? To address these issues the perceived effectiveness of 14 anti-doping measures was surveyed among 146 top athletes from Germany (Cycling: *N* = 42; Athletics: *N* = 104) who are members of at least the National Testing Pool. Results reveal significant differences in the perceived effectiveness of the anti-doping measures. Improved diagnostics were considered to be the most effective remedy for doping, followed by increased bans and the implementation of an anti-doping law. In contrast, fines and a leniency program were considered significantly less effective. Second, with the exception of indirect detection methods and increased use of an Anti-Doping Administration and Management System, results were consistent across cyclists and track and field athletes. Third, no significant gender difference was observed. Finally, an exploratory factor analysis showed that all anti-doping measures can be classified into the three categories *risk of detection* (e.g., control frequency and efficiency), *punishment* (e.g., fines and bans) and *communication* (e.g., education program). The results of this study provide a guideline for future research and for anti-doping and sport organizations when developing strategies against doping and allocating their anti-doping budget.

## Introduction

Barely a month passes without a headline-grabbing doping case. To fight doping and to ensure sports integrity the World Anti-Doping Agency (WADA) was founded in 1999 (World Anti-Doping Agency [WADA], 2017a). WADA’s anti-doping policy is specified in the World Anti-Doping Code (WADC), which defines doping “[…] as the occurrence of one or more of the anti-doping rule violations set forth in Article 2.1 through Article 2.10 of the *Code*” (World Anti-Doping Agency [WADA], 2015a, p. 18). Among others, such rule violations are the (attempted) use of prohibited substances or methods, evading sample collection or whereabouts failure. Although several activities against doping have been established since WADA was founded, doping is still one of the biggest issues the competitive sports’ world has to face.

The dimension of this problem becomes visible when comparing the numbers. The suspected amount of doping differs tremendously from the official anti-doping figures, which has lingered at approximately 1% for years (World Anti-Doping Agency [WADA], 2017b). In a review, the problem of estimating the prevalence of doping in elite sport was highlighted, as the estimates vary greatly depending on the method used (De Hon et al., 2015). Within the framework of population estimates based on biological parameters a prevalence rate of about 14% was estimated (Sottas et al., 2011), while standard questionnaires show rates from 1 to 15% (Laure, 1997) and the application of Randomized Response Technique (RRT) even shows estimates of 10–62% (Pitsch et al., 2007; Pitsch and Emrich, 2011; Ulrich et al., 2017).

These findings suggest that the present fight against doping is inefficient and that anti-doping measures must be reconsidered. In doing so, not only economic costs but also the athletes’ perception of those measures need to be considered. Athletes have to deal with various obligations that can affect their personal rights substantially. This is not only true for those athletes that might use illicit substances, but for all athletes. Since athletes are expected to know the true amount of doping best (most studies specifically ask athletes for their opinion), they should also be able to estimate the effectiveness of anti-doping measures. Therefore, and because anti-doping policy is developed “to protect the athletes’ fundamental right to participate in doping-free sport and thus promote health, fairness and equality for athletes worldwide” (World Anti-Doping Agency [WADA], 2015a, p. 11), it is of great importance to regard the athletes’ view.

## Anti-Doping Policy and its Background

Doping in elite sports has a long history and has become one of the biggest threats for sport itself over time (Houlihan, 2002). Hence, it is not surprising that more and more studies should investigate anti-doping policy and in particular the athlete’s decision to dope. Of great importance in this respect are inductively derived models of athletes’ doping behavior that have been developed based on a social cognition approach (Donovan et al., 2002), criminal deterrence theory (Strelan and Boeckmann, 2003), sports psychological constructs of goal directed behavior (Petróczi and Aidman, 2008) or a combination of these three (Mazanov and Huybers, 2010). A number of more specific studies focus on personal and psychosocial predictors of doping intention, or rather, use [for a meta-analysis see Ntoumanis et al. (2014)], and thereby largely rely on the theory of planned behavior (Ajzen, 1991).

Despite the increasing efforts to promote education on anti-doping, WADA’s policy still relies heavily on the deterrent effects of doping controls (Engelberg et al., 2015). The basic idea of such an anti-doping policy is that athletes perceive a high risk of detection (e.g., through high testing frequency or more effective diagnostics) and severe punishment (e.g., through long bans or high fines), and therefore will be less likely to engage in doping behavior (British Medical Association, 2002). Such an approach to understanding compliance with the law is in criminological research known as deterrence theory (Matthews and Agnew, 2008; Paternoster, 2010). This theory is closely related to the more general theory of planned behavior in that it suggests that when individuals contemplate committing a crime they weigh up the benefits and costs of doing so (Cornish and Clarke, 1986; Paternoster, 1987). Beside the general criticism of the deterrence theory and limited empirical support (Paternoster and Iovanni, 1986; Pratt et al., 2006), the main reason deterrence theory may have failed to explain appropriately the fight against doping is the low perceived risk of detection (Ayotte et al., 2013; Moston et al., 2015).

Due to the high number of unrecorded doping cases in elite sports over the last decades, it is impossible to calculate the effectiveness of anti-doping measures because of a missing reliable key indicator (De Hon et al., 2015). In the light of this and the insufficient deterrence by the current anti-doping regime, it is crucial to involve athletes in the development of the anti-doping work, considering that they may support the anti-doping system as a whole and that the protection of athletes is the main goal of WADA policy (Valkenburg et al., 2014; World Anti-Doping Agency [WADA], 2015a; Overbye, 2016a).

### Measures to Combat Doping

Shortly after the foundation of WADA in 1999 but years before the introduction of the first WADC in 2004 (World Anti-Doping Agency [WADA], 2015a), Striegel et al. (2002) saw the need for the inclusion and active participation of the athletes themselves to improve anti-doping measures. For that reason, they conducted a survey among German athletes who were subject to national and international anti-doping tests. From the athlete’s perspective improved detection methods and the provision of more information on the health risks were favored, followed by more frequent testing, whilst more severe punishment was not supported. In the following years, the WADC was periodically updated and several studies focused on the effectiveness of concrete anti-doping measures, whilst others looked at the adequacy of the anti-doping system as a whole.

Interview studies on doping abusers found that these athletes did not perceive the existing detection efforts in their sport as credible threats to deterring doping (Kirby et al., 2011; Pappa and Kennedy, 2012; Engelberg et al., 2015). A quantitative study among 488 elite athletes and 92 coaches showed moderate satisfaction with the current anti-doping regime, whereby a difference between these groups is reported: 62.9% of athletes and 47.8% of coaches “agreed or strongly agreed” with current measures (Moston et al., 2015). In a study with 645 participant Danish elite athletes, two-thirds of the participants agreed that the national testing program was appropriate (Overbye, 2016a).

There is, meanwhile, a number of studies investigating the perception of athletes regarding particular anti-doping measures. But up to now no study has compared a substantial number of them and there is no comprehensive overview of these measures. Based on the background of the current anti-doping regime, which is based in particular on deterrent measures, two categories of measures can be derived. On the one hand are measures that define the risk of detection from doping controls and on the other hand are measures that serve to punish convicted doping offenders. Since the anti-doping fight is becoming more and more complex, the provision of specific information is becoming increasingly important, so that *information-based measures* can be derived as a third category of anti-doping measures. On the basis of the three deduced categories of measures, the recent state of research is presented in the following.

### Risk of Detection

The probability of detection can be enhanced by increasing the control frequency, adopting more specific testing (follow-up testing or testing at night), and by using refined (improved diagnostics) or innovative detection techniques (indirect detection methods) to increase the probability of detection per test.

#### Control Frequency

To achieve the goals recorded in the WADC, adequate doping testing is a key measure (Overbye, 2016b). This is also reflected by the increasing number of tests conducted over recent years, which lead to 300,565 analyzed samples in 2016 by WADA-accredited laboratories (World Anti-Doping Agency [WADA], 2017b). This trend in testing figures follows the desire for more frequent drug testing expressed by athletes, despite the extensive invasion of the elite-athletes’ privacy (Striegel et al., 2002; Sas-Nowosielski and Swiatkowski, 2007; Overbye, 2016a). In spite of the official detection rate of around 1% many athletes still regard doping tests as a deterrent (Waddington et al., 2005; Dunn et al., 2010; Overbye, 2016b), whereby athletes with experience of testing within the past year were more likely to regard the likelihood of being selected for testing as a deterrent and only 40% of them found the risk of being selected for doping tests to be a deterrent (Overbye, 2016a). Using scenario analysis Strelan and Boeckmann (2006) show that doping testing has only a small influence on athletes’ drug use decisions.

#### Diagnostics

Every year the WADA releases an updated list of prohibited substance classes and methods (World Anti-Doping Agency [WADA], 2016a). Due to the progressive development of new doping substances and methods, many are not detectable at all or, at best, for a very short time period only (Houlihan, 2002; Pitsch, 2009; Ashenden et al., 2011; Lundby et al., 2012; Ayotte et al., 2013). Because of this difficulty and the great expense of the development of new detection methods and the consequent execution of new techniques, the proportion of positive doping tests has not increased (Overbye, 2016a), so that diagnosis has come to a “[…] race between doping and anti-doping” (Mottram, 2011, p. 31). Even most athletes are aware of this issue and support improved methods of detection (Striegel et al., 2002).

#### Indirect Detection Methods

In order to improve the testing regime, WADA introduced the Athlete Biological Passport (ABP) so that abnormal variations in specific biological values can be monitored (World Anti-Doping Agency [WADA], 2016b). The ABP can be used to identify athletes to target testing and may be used to pursue an anti-doping rule violation while the specific illicit substance no longer has to be to be detected itself (World Anti-Doping Agency [WADA], 2016b). Actually, the ABP just seems to keep doping within a certain limit, because strategic doping – like the use of micro-dosing – is difficult to prove even with the ABP (Ashenden et al., 2011; Lundby et al., 2012). In some countries like Germany, the ABP has already been implemented by the national anti-doping authority (Siekmann and Soek, 2010).

#### Follow-Up Testing

To bridge the gap between the use of novel doping substances and methods and their detection, follow-up testing has been established by WADA so that doping samples can be stored and tested up to 10 years after a sample is taken (World Anti-Doping Agency [WADA], 2015a). After re-testing around 1100 of samples collected during the 2008 Beijing and 2012 London Olympics, about 100 athletes got caught doping in this way in the period up to 2017 and not less than 75 medals have been withdrawn (International Olympic Committee [IOC], 2017). Surprisingly and despite the high chance of detection in the future, athletes regard follow-up controls as having only half of the deterrence effect compared to the risk of being caught doping shortly after sample collection (Huybers and Mazanov, 2012).

#### Testing at Night

Since some prohibited substances or methods have very short detection windows (Ashenden et al., 2011; Lundby et al., 2012), WADA introduced out-of-competition controls also between 11 p.m. and 6 a.m., so that athletes can be tested 24/7 on 365 days a year (World Anti-Doping Agency [WADA], 2015a). This leads to immense personal costs for the athletes, especially in terms of privacy (Valkenburg et al., 2014).

### Punishment

In order to punish athletes convicted of doping, fines and bans have become well-established measures, where the level of enforcement is much discussed and often differs in practice. Bans go beyond the financial dimension of fines by not only affecting future income from the (temporary) prohibition of following a profession, but also by influencing the athlete’s sporting career in the long term. An even tougher punishment goes along with an anti-doping law in some countries, as doping athletes have to fear imprisonment.

#### Fines

As mentioned before, the implementation of fines as a form of legal sanction is closely related to deterrence theory (Strelan and Boeckmann, 2003). It is not clear how effective athletes regard fines to be in combating doping. Some athletes regard fines as an ineffective deterrent (Engelberg et al., 2015) or do not favor fines (Striegel et al., 2002), while in other studies the majority of athletes were satisfied with the current fine policy (Waddington et al., 2005; Dunn et al., 2010). Huybers and Mazanov (2012) show that higher fines lead to a stronger deterrence effect, while within a scenario analysis material loss reaches the highest deterrence effect (Strelan and Boeckmann, 2006).

#### Bans

According to the WADC, athletes that violate the anti-doping rules get banned for up to 4 years for their first rule violation, while for a second violation a lifetime ban will be considered (World Anti-Doping Agency [WADA], 2015a). Although bans have severe consequences that may lead to the sudden end of a career, studies show that these possibilities are not assessed as highly probable by athletes (Huybers and Mazanov, 2012; Engelberg et al., 2015; Overbye, 2016b).

#### Anti-doping Law

Another opportunity is the criminalization of doping by a national law, because a criminal record with potential imprisonment may act as a more powerful deterrent than sanctions imposed by WADA or a sporting federation (World Anti-Doping Agency [WADA], 2015b; Sumner, 2017). As the number of countries with an anti-doping law grows, the way penalties are executed differs to some extent (Siekmann and Soek, 2010). A series of studies discuss the related advantages and disadvantages of such a state regulation (Zaksaite and Radke, 2014; Ioannidis, 2010; Sumner, 2017). Athletes’ views on the criminalization of doping are ambivalent even though the majority support the introduction of an anti-doping law (Striegel et al., 2002; Moston et al., 2015), but according to scenario analysis, criminalization possesses only weak deterrent properties (Strelan and Boeckmann, 2006).

### Information-Based Measures

In order to organize the multitude of annual doping tests, an appropriate data management system such as the Anti-Doping Administration and Management System (ADAMS) is indispensable. In addition, the anti-doping regulations are revised annually so that it is very important to provide athletes with the most up-to-date information. In order to draw the athletes’ attention to the negative consequences of doping at an early stage, a corresponding educational program is also indispensable. Finally, a leniency program could also be useful to obtain important background information on the use of doping so that it can be effectively combated.

#### ADAMS

In 2005, WADA established the Anti-Doping ADAMS (World Anti-Doping Agency [WADA], 2015a, 2017c). This Web-based database management tool has four primary functions: (1) Athlete Whereabouts, (2) Information Clearinghouse, (3) Doping Control Platform, and (4) Therapeutic Use Exemptions (TUE) Management (World Anti-Doping Agency [WADA], 2017c). Since elite athletes have to report, for every day in the forthcoming quarter, where they will sleep, train, and compete, in order to be located for out-of-competition testing at any time, this aspect is the most important one from the athletes’ perspective in terms of privacy (Dikic et al., 2011; Overbye and Wagner, 2014). The majority of athletes report that the Whereabouts system is important in detecting dopers (Valkenburg et al., 2014), and they perceive the system as necessary in order to carry out anti-doping tests effective and efficiently (Haristad et al., 2009; Overbye and Wagner, 2014). But on the other hand three quarter consider reporting Whereabouts as too time-consuming, 41% feel a reduced joy of being an elite athlete, and 22% feel under suspicion (Overbye and Wagner, 2014). As a result, the whereabouts system is criticized by a number of prominent athletes (Hanstad and Loland, 2009; Waddington, 2010; Møller, 2011). In terms of the technical aspects of the system, 69% of elite athletes have confidence in these, while 19% do not (Haristad et al., 2009). Subsequently, the *ADAMS-App* was introduced to make Whereabouts management more convenient for athletes (World Anti-Doping Agency [WADA], 2014), but this feature has not been considered in any study so far.

#### Provision of Anti-doping Rules

Through the years, the Anti-Doping rules and therefore also the demands on the athletes have become increasingly complex, making it necessary to provide the information on anti-doping noted in WADC to the athletes in an appropriate way. Studies have shown that there is an immense lack of knowledge on the part of athletes regarding the current anti-doping rules (Waddington et al., 2005; Sas-Nowosielski and Swiatkowski, 2007). It is notable, that young adults have more knowledge in terms of anti-doping compared to older athletes (Sas-Nowosielski and Swiatkowski, 2007). Whereas, some years ago the most frequently indicated source of knowledge was television followed by the internet, peers, coach, and sport press (Sas-Nowosielski and Swiatkowski, 2007), the role of the internet has become more important due to digitalization, whereby sources of information like Apps (i.e., *WADA-App* or *NADA-App* in Germany) has become more important.

#### Education Program

Not only the knowledge deficit outlined above emphasizes the necessity of coordinated education programs. The main purpose of anti-doping education programs is to change the athletes’ attitudes toward doping. Consequently these programs can be assigned to at least one of three traditional approaches to anti-doping education (Lucidi et al., 2017). Firstly, the “scared-based” approach focuses mainly on negative health risks, but has proven inefficient so far due to its possible boomerang effect (Goldberg et al., 1991); for a review see Petróczi et al. (2014). Secondly, specific training programs focus on ethical decision making that can be positively affected by ethics-based education (Elbe and Brand, 2016). Thirdly, knowledge-based approaches show divergent results (Fritz et al., 2005; Goldberg and Elliot, 2005; Elliot et al., 2008; Backhouse et al., 2014). Based on the theory of planned behavior, Ntoumanis et al. (2014) showed that a knowledge-based education approach reduces the athletes intention to dope, but does not result in a change in doping behavior. A study on media literacy intervention showed that such measures are effective in decreasing adolescent’s positive attitudes toward doping (Lucidi et al., 2017).

#### Leniency Program

A number of headline-grabbing doping scandals like the Lance Armstrong case or the scandal concerning systematic doping in Russian Athletics have been revealed by whistleblowers (Heberling, 2014). The revised WADC (Article 10.6.1) supports the provision by athletes of substantial assistance to uncover anti-doping rule violations by offering the possibility of reducing the length of their sanctions reduced or even completely remove them (World Anti-Doping Agency [WADA], 2015a). Up until now there have been some studies with a focus on athletes’ perception of reporting doping or whistleblowing (Whitaker et al., 2014; Erickson et al., 2017), but none take account of a leniency program.

## This Study

It could be shown that there is ample but unstructured literature about different measures to combat doping. However, a number of these studies are very outdated, as there have been some fundamental changes in anti-doping policy since the 2002 study by Striegel et al. (2002), for example, so that a current study is necessary. Additionally, there are hardly any studies that examine the effectiveness of the measures and none that compare several measures with regard to their effectiveness. But it is precisely these findings that are of crucial importance for the efficient further development of the fight against doping.

In view of the fact that the effectiveness of the measures cannot currently be measured directly, since the true prevalence of doping cannot be measured with sufficient precision, other methods must be chosen. Therefore, it appears eminently reasonable to ask athletes about the perceived effectiveness of the various measures, as the different measures are intended for the athletes and they therefore should be able to assess them best. It must be noted that the results of the survey do not reflect the objective effectiveness of a given measure, but the perceived effectiveness of the measures influences the doping decision of the athletes and is therefore of great importance. In order to address this substantial research gap, a study was conducted among top athletes to answer the following research questions (RQ):

RQ1: How effective do top-level athletes of different sports perceive applied anti-doping measures to be?

In addition, it is not yet clear whether athletes from different sports perceive anti-doping measures as equally effective and whether this perception is stable across the genders. Against this background, the following RQ will be answered:

RQ2: Do athletes from different sports consider the effectiveness of the various anti-doping measures to be equally effective?
RQ3: Do male and female athletes perceive the effectiveness of the various anti-doping measures as equally effective?

The literature review on anti-doping measures presented above was derived from theory or was logically motivated. However, there is no empirical evidence for categorizing the measures, which could be of great benefit for further research. In order to make a valuable contribution to the research on anti-doping policy, the following research question will be answered:

RQ4: How can the anti-doping measures examined be divided into appropriate empirical categories of measures regarding their perceived effectiveness?

To answer the four RQ raised, a quantitative survey was conducted among top athletes who have experience with the current anti-doping regime. For this reason, cyclists and athletes were surveyed, as both sports belong to the highest risk group of the German National Anti-Doping Agency (NADA) (National Automobile Dealers Association [NADA], 2016) and no sport in this risk group is subjected to more doping controls each year (World Anti-Doping Agency [WADA], 2017b). It can therefore be assumed that these two groups of athletes have the most extensive experience with the current anti-doping regime. Moreover, both sports have repeatedly been the focus of past doping scandals.

## Materials and Methods

### Sample

A total of 146 top athletes took part in our study. Of these athletes 42 were professional cyclists from Germany and 104 were track and field athletes belonging to the current German track and field national squad. While all cyclists were male, 44 of the 104 of the track and field athletes were female (42%). Furthermore, the track and field athletes are subdivided into five discipline classes. Of the 104 track and field athletes 30 were sprinters, 35 middle or long distance runner, 19 jumpers, 14 throwers, and six were combined event athletes. Due to anonymity issues no other personal information such as age, residence or team membership was sought. The data collection was carried out in collaboration with the German Cycling Federation and the Track and Field Federation. The Cycling Federation sent the participant information and the link to the survey to all male professional license holders, members of the U23 and elite national squads, while the Track and Field Federation sent the same information to all male and female elite squad athletes, so that a self-selected sample is given here. This process ensured that at the time of the survey, all participants were at least 18 years old, still active in their sport, and therefore part of at least the National Testing Pool. Thus, personal experiences with the recent anti-doping policy can be assumed. Participation was voluntary and the questionnaire could be canceled at any time. The study design and procedure as well as handling of data were ethically approved by the ethics committee of the Faculty Psychology and Sport and Exercise Sciences of the University of Muenster. Cyclists participated in April and May 2015 and the track and field athletes participated in April and May 2016.

### Measures

This study used a structured online questionnaire with 14 questions. One question differed between the two groups of athletes (for questionnaire see **Appendix A**). The participating athletes were asked to assess selected anti-doping measures on a five point Likert scale, which ranged from “not effective” (1) to “very effective” (5). Since the original version of the applied questionnaire was designed in German and is now used for publication in English, a back-translation method was used (Hambleton, 2001). Next to the results presented here, we queried further variables. This study is part of a larger research project, so that besides the variables listed here, other variables about the perceived trustworthiness of selected actors in sport and doping behavior via RRT were collected.

### Data Analysis

Data was calculated by using SPSS Software (Version 24). Paired and 2-tailed *t*-tests were measured. Alpha level was set at *p* < 0.05.

### Descriptive Statistics

To address research question 1, the mean values and standard deviations (SD) of the evaluated anti-doping measures were calculated. In **Table 1**, anti-doping measures are sorted by means of total sample in descending order. The mean ranged from 2.726 (*Increased use of ADAMS*) to 4.288 (*Improved diagnostics*). Means and SD are also reported separately for Athletics and Cycling and disparities in means between these two groups are also calculated. Small differences are reported in particular for *education program*, while *increased use of ADAMS* shows by far the highest disparity.

**Table 1:** Evaluation of selected anti-doping measures by top-level athletes.

	Mean (*N* = 146)	SD	Athletics (*N* = 104)	SD	Cycling (*N* = 42)	SD	Disparity in mean
Improved diagnostics	4.288	0.879	4.250	0.943	4.381	0.697	–0.131
Increase of bans	4.007	1.105	4.058	1.113	3.881	1.087	0.177
Anti-doping law	3.747	1.225	3.625	1.286	4.048	1.011	–0.423
More follow-up controls	3.726	1.177	3.673	1.218	3.857	1.072	–0.184
Indirect detection methods	3.712	1.017	3.558	1.022	4.095	0.906	–0.538
Increase of control frequency	3.630	1.089	3.481	1.106	4.000	0.963	–0.519
Education program	3.555	1.157	3.538	1.165	3.595	1.149	–0.057
Provision of anti-doping rules	3.390	1.206	3.317	1.248	3.571	1.085	–0.254
Increase of fines	3.247	1.195	3.192	1.133	3.381	1.343	–0.189
Use of NADA app	3.205	1.259	3.087	1.263	3.500	1.215	–0.413
Leniency program	3.000	1.057	3.048	1.018	2.881	1.152	0.167
Increased use of ADAMS	2.726	1.235	2.471	1.222	3.357	1.032	–0.886
Testing at night (11 p.m.–6 a.m.)					3.738	1.398	
Use of ADAMS app			3.099	1.338			

At this point it should be noted that the item *Use of ADAMS App* was presented only to the track and field athletes, while *Testing at night* was presented only to the cyclists, so that these measures are not taken into account in the further analysis. In the next section, the disparities between cyclists and track and field athletes are analyzed in more detail.

### Comparative Analysis of Cycling and Athletics

For testing the significance of differences between cyclists and track and field athletes in perceived effectiveness of the anti-doping measures considered and therefore addressing research question 2, an independent-samples *t*-test was conducted. In **Table 2**, the *t*- and *p*-value as well as effect size (Cohen’s *d*), lower and upper confidence interval (95%) are reported. The correlation matrix of the measures considered is provided in **Appendix B**.

**Table 2:** Comparative analysis of selected anti-doping measures.

Anti-doping measure	t	*df*	*p*-value	Cohen’s d	95% CI
Improved diagnostics	–0.924	101.846	0.358	0.148	(–0.412, 0.150)
Increase of bans	0.883	77.582	0.380	0.163	(–0.222, 0.575)
Anti-doping law	–2.107	95.816	0.038	0.346	(–0.821, –0.024)
More follow-up controls	–0.902	85.628	0.370	0.161	(–0.590, 0.222)
Indirect detection methods	–3.126	85.099	0.002ˆ**	0.545	(–0.879, –0.196)
Increase of control frequency	–2.823	86.554	0.006	0.487	(–0.885, –0.154)
Education program	–0.269	76.858	0.788	0.052	(–0.477, 0.363)
Provision of anti-doping rules	–1.225	86.679	0.224	0.208	(–0.666, 0.158)
Increase of fines	–0.803	65.795	0.425	0.159	(–0.658, 0.281)
Use of NADA app	–1.841	78.628	0.069	0.328	(–0.861, 0.034)
Leniency program	0.820	68.244	0.415	0.161	(–0.240, 0.574)
Increased use of ADAMS	–4.446	89.247	0.001ˆ***	0.760	(–0.282, –0.490)

*^∗^*p* < 0.05. ^∗∗^*p* < 0.01. ^∗∗∗^*p* < 0.001.*

Based on Levene’s test for homoscedasticity, equal variances are not assumed. Due to the issue of multiple comparisons within the performed *t*-test Bonferroni-Holm correction (Holm, 1979; Armstrong, 2014) was used to adjust observed significance reported in **Table 2**. After Bonferroni-Holm correction, there was a significant difference in the scores for *indirect detection methods* between track and field athletes (M = 3.558, SD = 1.022) and cyclists (M = 4.095, SD = 0.906); *t*(85.099) = -3.126, *p* = 0.002. Another significant difference was found for *increased use of ADAMS* between track and field athletes (M = 2.471, SD = 1.222) and cyclists (M = 3.357, SD = 1.032); *t*(89.247) = -4.446, *p* ≤ 0.001. Both significant observations show a large effect with Cohen’s d magnitudes of 0.545 and 0.760, respectively. Bonferroni-Holm correction did not show significant effects for *criminal prosecution* and *increase of control frequency* despite comparatively low *p*-values of 0.038 and 0.006, respectively. All other anti-doping measures considered did not show significant differences.

### Comparative Analysis of Gender Differences

To address research question 3, the next step was to check whether gender differences exist. Since only the track and field group was made up of men and women, the second independent-samples *t*-test contains only 104 athletes. All relevant information is presented in **Table 3**.

**Table 3:** Comparative analysis of gender differences.

Anti-doping measure	t	*df*	*p*-value	Cohen’s d	95% CI
Improved diagnostics	0	85.734	1	0	(–0.381, 0.381)
Increase of bans	0.266	82.694	0.791	0.054	(–0.393, 0.514)
Anti-doping law	0.078	98.121	0.938	0.015	(–0.480, 0.520)
More follow-up controls	–0.716	94.474	0.476	0.139	(–0.652, 0.306)
Indirect detection methods	–0.469	87.389	0.640	0.088	(–0.508, 0.314)
Increase of control frequency	–0.689	92.866	0.493	0.135	(–0.588, 0.285)
Education program	0.978	95.619	0.331	0.189	(–0.231, 0.679)
Provision of anti-doping rules	–0.817	98.937	0.416	0.152	(–0.681, 0.284)
Increase of fines	–0.436	87.103	0.664	0.088	(–0.556, 0.356)
Use of NADA App	–0.960	87.867	0.340	0.198	(–0.749, 0.261)
Leniency program	0.214	88.614	0.831	0.049	(–0.364, 0.452)
Increased use of ADAMS	–1.153	83.594	0.252	0.238	(–0.780, 0.207)

*^∗^*p* < 0.05. ^∗∗^*p* < 0.01. ^∗∗∗^*p* < 0.001.*

Again, based on the level test for homoscedasticity, no equivalent deviations are assumed. In comparison to the previous *t*-test, no significant differences could be found here. Since the Bonferroni-Holm correction is a conservative form to correct the alpha error, it was not carried out here. Therefore, it can be concluded that there are no significant gender differences in assessing the effectiveness of anti-doping measures.

### Categorization and Comparison of Anti-doping Measures

For research question 4, exploratory factor analysis (EFA) via principal components analysis using varimax rotation was performed to identify the underlying factor structure of the current anti-doping regime in top-level sports. The 12 variables used for comparative analysis and 146 cases were entered into the analysis giving a variable to subject ratio of 1:12.17. Whilst advice regarding sample size for EFA varies, Kline (1994) recommends variable-to-subject ratios of at least 1:5.

Initially, the factorability was examined by using several well-recognized criteria. It was observed that 11 of the 12 items had a correlation of at least 0.3 with at least one other item (see **Appendix B**). After removing the item *leniency program* that showed no correlation above 0.3, a reasonable factorability was suggested. In the next step, the Kaiser-Meyer-Olkin measure of sampling adequacy was 0.648 and thus above the recommended value of 0.6. Bartlett’s test of sphericity was significant [χ^2^ (55) = 291.506, *p* < 0.05]. The diagonals of the anti-image correlation matrix were also all above 0.5. Finally, the communalities were all above 0.3 (see **Table 4**), further confirming that each item shared some common variance with other items. Given these overall indicators, factor analysis seemed to be suitable for the remaining eleven items.

**Table 4:** Factor loadings and communalities based on a principal components analysis for 11 items (*N* = 146).

Factor	1	2	3	Communalities
***(1) Risk of Detection***				
Increased use of ADAMS	(0.710)	0.071	0.048	0.512
Increase of control frequency	(0.628)	–0.054	–0.211	0.442
Increased use of indirect detection methods	(0.626)	–0.248	–0.234	0.508
Improved diagnostics	(0.506)	–0.287	–0.502	0.591
More follow-up controls	(0.450)	–0.086	–0.549	0.512
***(2) Communication***				
Provision of recent anti-doping rules	0.400	(0.728)	0.077	0.695
Education program	0.364	(0.607)	0.190	0.538
Use of NADA App	0.401	(0.481)	0.267	0.463
***(3) Punishment***				
Increase of fines	0.459	–0.237	(0.574)	0.596
Increase of bans	0.306	–0.333	(0.472)	0.427
Anti-doping law	0.289	–0.595	(0.455)	0.644
Eigenvalues	2.591	1.809	1.526	

A three latent factor solution with simple structure was identified. Extraction of factors was based upon Kaiser’s criterion for Eigenvalues of equal or greater than unity. The three factors identified, compromising eleven items, accounted for 53.88% of the total variance within the data.

The first factor is labeled *Risk of Detection* (mean = 3.38) and encompasses five items, namely *increased use of ADAMS, increase of control frequency, Indirect detection methods, Improved diagnostics*, and *more follow-up controls*. Hence, it covers administrative as well as qualitative and quantitative aspects of anti-doping testing. The factor *Risk of Detection* accounted for 23.55% of the total variance and had an Eigenvalue of 2.591. General factor saturation was determined by Cronbach’s alpha and is 0.60 for this factor.

The second factor, which is labeled *Communication* (mean = 3.62), comprises the items *education program, provision of recent anti-doping rules*, and *use of NADA App.* Therefore this factor covers value and information-based education and its dissemination. It accounts for 16.45% of total variance and has an Eigenvalue of 1.809. Cronbach’s alpha is 0.643.

Lastly, the third factor, labeled *Punishment* (mean = 3.76), covers punishments by sporting federations and governments. This factor comprises the three items *increase of fines, anti-doping law*, and *increase of bans* and accounted for 13.88% of the variance. It has an Eigenvalue of 1.526 and Cronbach’s alpha for this factor is 0.601.

All items in the analysis have primary loadings and communalities over 0.4. The factor loading matrix and communalities are presented in **Table 4**. No increases in alpha for any of the factors have been achieved by eliminating more items.

At this point no EFA was carried out for individual sports because the group of cyclists (*N* = 42) is too small and, also, for this group the variable-to-subject ratio of 3.818 is considerably lower than the required value of 5.

## Discussion

Based on this study, valuable implications for anti-doping research and policy can be deduced. Although only German top athletes have taken part in the study, the results and recommendations for action can be applied on the international level, as the athletes surveyed also compete at this level. The extent to which the results can be transferred from one country to another remains to be verified, as some aspects of the WADC are implemented at the national level in very different ways. Moreover, it is precisely because of athletes’ test pool membership that extensive experience with anti-doping policies can be assumed, so that doping controls are not just a theoretical procedure for these athletes.

In the context of RQ1, it was shown for the first time that top athletes perceived some of the anti-doping measures examined as being very different in terms of their effectiveness. Apart from two exceptions, there was no significant difference in the perception of cyclists and track and field athletes (RQ2). Moreover, the results were stable across both genders (RQ3). A special contribution was made by categorizing the anti-doping measures, which were considered for the first time on an empirical basis (RQ4). The categories discovered here are similar in content to the previously derived categories, but the assignment of measures differs in some respects. In the following, the measures investigated are discussed according to this categorization, with a particular focus on perceived effectiveness and any differences between sports.

A number of studies have criticized WADA’s policy for focusing on deterrence rather than on education strategies to combat doping in sport (Engelberg et al., 2015; Moston et al., 2015). The results of this study suggest that athletes consider communication measures like education programs to be reasonable, but stricter controls and harsher punishment to be a more effective means to fight doping in sport. It is therefore questionable if the deterrence effect of doping testing and punishments are generally more meaningful measures to deter athletes from doping behavior or if it is more likely that the current communication actions are not implemented appropriately to reach the athletes effectively.

### Risk of Detection Measures

When looking at risk of detection measures, it has to be emphasized that athletes regard a higher detection probability through improved diagnostics to be the most effective anti-doping measure. It is therefore reasonable that athletes perceive the number of undetected doped athletes to be very high or even know doping athletes that pass doping tests on a regular basis. Due to the immense progress in medical science new doping substances and methods are developed continuously, so that it is of great importance to invest in new and more sensitive detection methods.

Along similar lines, the participating athletes regard the more frequent use of follow-up controls or indirect detection methods to be important for an effective anti-doping policy. Cyclists and track and field athletes have a similar view on follow-up testing, which might be the result of the large number of headline-grabbing cases in both sports. In contrast, these athletes have strong and significantly different views on indirect detection methods like ABP. The reason may be that the ABP was introduced in cycling first and a number of popular riders were caught doping (Brown, 2009; Cycling News, 2010). As a consequence some kind of a realistic deterrence effect might have taken shape, because high-level riders perceive the risk of being caught to be high and may fear target testing based on ABP. In comparison, ABP was introduced later by the International Association of Athletics Federations (IAAF), but despite a series of striking ABP values a comparatively small number of athletes has been convicted by IAAF so far (International Association of Athletics Federations [IAAF], 2010; Sottas et al., 2011; The Sunday Times, 2015). The implementation of the ABP led to a large decrease in the percentage of abnormal blood parameters among professional riders, while in Athletics the decrease is more moderate (The Sunday Times, 2015; Zorzoli, 2011). It therefore can be concluded that indirect detection methods, particularly in cycling, are seen as a deterrent and have led to changes in behavior, whilst in Athletics this process started some years later. In the future, ABP should be developed in a way that supports the indirect detection of new doping substances and methods or target testing. Moreover, the communication of its power could be crucial to achieve stronger deterrence.

In this context, the implementation of testing between 11 p.m. and 6 a.m. is discussed frequently, due its potential to overcome the short detection windows of some doping practices. That cyclists perceive it as moderately effective might be based on privacy issues and that testing at night could become a competitive disadvantage, because of disturbing athletes’ regular recovery process. Nevertheless, cyclists seem to see the benefits of testing at night due to the fact that some doping practices are actually detectable for less than 7 h, so that it could be a doping athletes’ strategy to use such practices at 11 p.m. Thus, against the backdrop of privacy and fairness issues this measure should be further discussed with athletes before being implemented on a regular basis. A solution could be to conduct tests at night primarily for target testing based on ABP.

A reason for the perception of increased control frequency as moderately effective could be that both groups of athletes perform in a sport that is categorized as “highly risky” by NADA Germany, leading to a relatively high control frequency so that further increases of testing frequency are perceived as unnecessary even with regard to privacy issues. Even though no significant differences could be found here, the absence of such difference could be explained by differences in control frequency among the sports under consideration. Official testing figures show that control frequencies reported by NADA Germany and WADA are higher in athletics than in cycling, respectively (National Automobile Dealers Association [NADA], 2017; World Anti-Doping Agency [WADA], 2017b).

Furthermore, both groups of athletes perceive the effectiveness of an increased use of ADAMS as very low. This may be a result of the time-consuming administration of athletes’ whereabouts, so that they do not want to report more information than is currently required. Also, doubts regarding privacy issues like lack of knowledge about where the ADAMS data are stored, who has access, and how they are used and secured may lead to this kind of mistrust in the ADAMS system as a whole. Although both groups of athletes have doubts concerning ADAMS, track and field athletes perceive ADAMS as significantly more ineffective than do cyclists. Apparently, the current version of the ADAMS App does not seem to have a positive impact on that view, while track and field athletes also perceive it as relatively ineffective. Against the backdrop of these results, it is important to develop the ADAMS system and the associated App with the help of the athletes. In doing so, it should be further discussed if GPS-positioning systems could partially replace reporting via ADAMS, because it would be less time consuming for athletes. Moreover, it is crucial to provide information about how ADAMS works, its data security and why athletes should support it.

### Punishment Measures

Measures categorized as punishment are generally perceived as effective, although differences exist. Cyclists and track and field athletes agree that an increase of bans is effective in deterring doping. However, it has to be considered that a further extension of bans practically amounts to a lifetime ban or, rather, an occupational ban. Therefore, a further extension has to be discussed especially on legal grounds. In any case, the imposition of bans should be more consistent across all nations and sports, in accordance with the WADC’s guidelines (4 years for first offenses, followed by lifetime bans for second offenses).

Compared to extended bans, increased fines are assessed as far less effective by both groups of athletes. As a consequence, fines can be seen as a reasonable complementary measure with a moderate deterrence effect so that not too much attention has to be paid compared to other measures. In that regard, it seems to be reasonable to reinvest the collected fines into more effective anti-doping measures like diagnostics or education programs.

The implementation of an anti-doping law was discussed as a very controversial topic in Germany before its adoption. Both groups of athletes perceive an anti-doping law as a valuable supplement. This may be because athletes consider that leading sporting bodies do not have the ability or even intention to fight doping appropriately. Such a state intervention leads to new ways of criminal investigation and new punishments. A further challenge will be to synchronize government and sporting bodies’ regulations.

### Communication Measures

Most of the communication measures are actually perceived as being less effective in keeping athletes from abuse doping than are control or punishment measures. Overall, education programs are perceived as moderately effective. This confirms that athletes may have a divergent view on such programs, depending on their focus. Therefore, education programs should be developed with the active involvement of the athletes resulting in age- or sports-specific education programs, for example. Due to the increasing importance of social media, in particular for young people, anti-doping authorities should endeavor to use different social media channels to make young athletes aware of doping issues.

The provision of anti-doping rules is also evaluated as moderately effective. Since further studies reveal a substantial lack of knowledge and confessed dopers regularly pretend that they were not aware of the detected prohibited substances, more effort is required to counteract the lack of awareness of anti-doping rules. Therefore, basic knowledge on anti-doping rules should be part of every education program and it must be outlined where athletes can find detailed information like the most recent anti-doping list or the responsible use of dietary supplements.

In this context, a mobile application like the NADA App can have increased importance. At the time of data collection, the recent anti-doping list was available via the NADA App. Beyond that only limited information was provided. It is thus not surprising that the NADA App is evaluated as less effective than most measures. From an athlete’s point of view, a useful enhancement could be the use of a barcode so that drugs and dietary supplements can be scanned in order to check whether their use is compliant with the anti-doping rules.

### Leniency Program

The final measure to discuss is the leniency program for confessed dopers. Although athletes actually perceive such a program as not very effective, it offers certain opportunities to combat doping effectively. Through the implementation of a reporting or rather whistleblowing system, investigating authorities can gather important information on doping behavior and can react, among other things, with appropriate target testing. In addition, this information is of special importance to develop better detection methods and anti-doping strategies, because otherwise anti-doping authorities would not know what to look for. The moderate evaluation of a leniency program could be partly explained by popular cases like that of the former professional cyclist Patrick Sinkewitz (Spiegel, 2008). After his first detected doping offense he made use of a leniency program and reported information on doping behavior to the authorities and consequently received a reduced ban. But instead of returning to professional cycling clean, he got caught doping again just shortly after his comeback. Consequently, it is not surprising that cyclists do not really believe in the effectiveness of such a leniency program. Referring to athletics, the survey providing data for this study was conducted before the popular and groundbreaking whistleblowing case of Yuliya and Vitaly Stepanov was made public, so that it can be assumed that athletes were not aware of the importance whistleblowing can have to uncover doping scandals (Brant, 2016). Therefore, it is important to communicate the immense potential benefits that a leniency program entails to make anti-doping policy effective. In doing so whistleblowing can become a fourth pillar of anti-doping policy beside control, communication and punishment. The results of the EFA supports that view, because leniency programs do not fit one of the three explored factors. When implementing whistleblowing systems, data security and anonymity are of great importance. In addition, it is necessary to clarify how concrete the indications must be or whether mere speculations are sufficient to open a case.

### Financial Aspects

The results of this study are useful to derive recommendations for anti-doping budget allocation. The budget of the NADA Germany amounted 9,651,085 € in 2016 (National Automobile Dealers Association [NADA], 2017) and is therefore very small compared with the sums invested annually in sport. With 34.2% most of the budget was spend for “doping testing,” followed by 22.2% for “research and analysis,” and 10.6% for “prevention projects.” Only 1.4% was spent for “communication and marketing.” By comparing current anti-doping budget allocation with the results of this study, a relatively high proportion of the budget is invested in doping testing. Instead, more of the budget should be invested in research on improved diagnostics and indirect detection methods. Furthermore, higher investment is required in sample analysis to conduct more follow-up tests. Further investment in education programs should also focus on digital content, due to its increasing importance. In light of the relatively low budget for communication and marketing, consideration should be given to raising this budget to increase public attention on anti-doping efforts and to receive more support through sponsorship, because in doing so NADA Germanys’ revenues can be increased.

### Limitations and Future Directions

The present study has some limitations. Firstly, the measures “testing at night” and “use of ADAMS” were not presented to both groups of athletes, so that conclusions are limited for these two measures. Secondly, it must be considered that the results are based on self-reported and subjective data, so that despite the importance of the athletes’ view on anti-doping policy, these data reflect only the perceived effectiveness of the anti-doping measures.

Due to the facts that only German athletes participated in this study and that anti-doping efforts vary widely from country to country, the results and implications of this study should be transferred to other countries with caution, although NADA Germany is one of the leading anti-doping agencies worldwide. Furthermore, a longitudinal study is desirable to adjust anti-doping policy in the future with respect to changing circumstances.

## Conclusion

In the course of this study among top-level cyclists (*N* = 42) and track and field athletes (*N* = 104), the measures to combat doping implemented by anti-doping authorities were divided into three reasonable pillars: Risk of Detection, Punishment, and Communication. Overall, all three pillars were considered by the athletes to be effective, with more stringent controls and harsher penalties assessed by the athletes as more effective than communication measures. Whistleblowing can also be understood as a fourth pillar of the fight against doping, which was perhaps covered by the assessment of the leniency program and could be interpreted as a further important research field. On measurement-level, both athlete groups rated improved diagnostics as the most effective anti-doping measure. Only indirect detection methods and increased use of ADAMS were found to be significantly more effective by cyclists than by track and field athletes. On the other hand, no gender differences could be identified across all evaluated measures. Based on these results, anti-doping organizations should rethink their policies and, if necessary, reallocate their investments.

## Ethics Statement

Ethics approval for the conduct of the study was granted by the “Faculty Research Ethics Committee, Faculty of Psychology and Sport & Exercise Sciences, University of Münster” with written informed consent from all subjects.

## Author Contributions

DW led the development and implementation of this research project. DW, DD, BS, and GS collaborated to develop and design the questionnaire. DW collected the data from the cyclists and JP the data from the track and field athletes. DW and DD conducted the data analysis. Afterward, all authors discussed the results. DW wrote the initial draft of the paper and all other authors provided comments on the initial draft and therefore contributed to the final manuscript.

## Conflict of Interest Statement

The authors declare that the research was conducted in the absence of any commercial or financial relationships that could be construed as a potential conflict of interest.

## Appendix

### A – Questionnaire

#### 1. Statements on the Anti-doping Measures

Below we would like to know from you, how you assess the work of the German national anti-doping agency (NADA).

Please rate, whether the following measures contribute to the fight of doping abuse. It is not about what exactly you know about the work, but about your feeling.

**Table A1:** Questionnaire.

	Not effective 1	2	3	4	Very effective 5
Increase of fines	O	O	O	O	O
Criminal prosecution (Anti-Doping-Law)	O	O	O	O	O
Increase of bans	O	O	O	O	O
Education program for young athletes	O	O	O	O	O
Provisioning of latest anti-doping-information (Prohibited List etc.)	O	O	O	O	O
Increase of control frequency	O	O	O	O	O
More frequent provision of follow up inspection of the samples	O	O	O	O	O
Improvement of the diagnosis for evidence of banned substances and methods	O	O	O	O	O
Expansion of the application of indirect detection methods (biological passport – ABP)	O	O	O	O	O
Increased application of ADAMS (Information system for contribution of Whereabouts, etc.)	O	O	O	O	O
Leniency program for admitting athletes	O	O	O	O	O
Use of the NADA App	O	O	O	O	O
Use of ADAMS App (Athletics only)	O	O	O	O	O
Testing at night between 11 p.m. and 6 a.m. (Cycling only)	O	O	O	O	O

### B – Correlations

**Table A2:** Correlation matrix.

Variables	1	2	3	4	5	6	7	8	9	10	11	12
1. Increase of fines	1											
2. Anti-doping law	0.354	1										
3. Increase of bans	0.338	0.312	1									
4. Education program	0.155	–0.143	0.035	1								
5. Provision of recent anti-doping rules	0.019	–0.217	0.008	0.442	1							
6. Increase of control frequency	0.171	0.105	0.077	0.137	0.190	1						
7. More follow-up controls	0.034	–0.044	0.012	0.122	0.032	0.265	1					
8. Improved diagnostics	0.004	0.068	0.161	–0.124	0.056	0.285	0.430	1				
9. Increased use of indirect detection methods	0.161	0.218	0.032	0.060	0.019	0.320	0.262	0.340	1			
10. Increased use of ADAMS	0.261	0.154	0.072	0.213	0.244	0.365	0.133	0.219	0.420	1		
11. Leniency program	0.240	0.091	0.177	0.135	0.179	–0.012	0.066	0.178	0.257	0.206	1	
12. Use of NADA App	0.122	0.034	0.049	0.238	0.447	0.046	0.024	0.040	0.073	0.263	0.275	1
